# Fear of Reinjury Following Anterior Cruciate Ligament Reconstruction Is Manifested in Muscle Activation Patterns of Single-Leg Side-Hop Landings

**DOI:** 10.1093/ptj/pzab218

**Published:** 2021-09-21

**Authors:** Jonas L Markström, Adam Grinberg, Charlotte K Häger

**Affiliations:** Department of Community Medicine and Rehabilitation, Physiotherapy, Umeå University, Umeå, Sweden; Department of Community Medicine and Rehabilitation, Physiotherapy, Umeå University, Umeå, Sweden; Department of Community Medicine and Rehabilitation, Physiotherapy, Umeå University, Umeå, Sweden

**Keywords:** Biomechanics, Electromyography, Knee Injuries

## Abstract

**Objective:**

The purpose of this study was to determine whether fear of re-injury is manifested in joint kinematics and muscle activation patterns during landings of a standardized rebound side-hop (SRSH), or in patient-reported outcome measures (PROMs), among individuals with anterior cruciate ligament reconstruction (ACLR).

**Methods:**

In this cross-sectional observational study, 38 individuals within 2 years post-ACLR were grouped into HIGH-FEAR (n = 21, median 11.2 months post-surgery) or LOW-FEAR (n = 17, median 10.1 months post-surgery) based on a discriminating question (Q9; Tampa Scale of Kinesiophobia-17). These individuals and 39 asymptomatic controls performed the SRSH. Three-dimensional motion recordings were used to calculate trunk, hip, and knee joint angles at initial contact and range of respective joint motion during landing. Surface electromyography registered mean amplitudes and co-contraction indexes for thigh muscles during pre-activation (50 ms) and landing phases. PROMs of knee function, knee health, and physical activity were also analyzed.

**Results:**

The HIGH-FEAR and LOW-FEAR classification was corroborated by distinct Tampa Scale of Kinesiophobia-17 total and subscale scores and revealed distinguishable muscle activation patterns. HIGH-FEAR demonstrated higher biceps femoris electromyography amplitude and higher anterior-posterior co-contraction index during landing than both LOW-FEAR and controls. However, there were no fear-related differences for kinematics or PROMs. Instead, both ACLR subgroups showed different kinematics at initial contact to controls; HIGH-FEAR with more trunk, hip, and knee flexion, and LOW-FEAR with more hip and knee flexion.

**Conclusion:**

Individuals with ACLR who had high fear of re-injury seem to have adopted a protective strategy with higher muscular activation patterns, presumably to stabilize the knee joint, compared with individuals with low fear of re-injury and controls. SRSH landing kinematics or knee-related PROMs may not be as sensitive to fear of re-injury.

**Impact:**

Fear of reinjury following anterior cruciate ligament injury should be evaluated as an independent psychological outcome throughout rehabilitation after ACLR for improved return to sport transition.

**Lay Summary:**

If you have an anterior cruciate ligament injury treated with reconstructive surgery, you might have a high fear of reinjury, and that can change how you activate the muscles around your knee. Your physical therapist can do a simple screening test in addition to functional tests to help reduce your fear and improve your treatment outcomes.

## Introduction

Rupture of the anterior cruciate ligament (ACL) is a devastating knee injury that often results in physical^1–4^ and psychological^5^^,^^6^ acute and chronic consequences. Roughly 70% of all ACL injuries occur during sports participation.^7^ Most of these injuries occur in situations of non-contact or indirect contact with momentarily poor movement control^8^^,^^9^ during rapid side-to-side movement maneuvers or single-leg landings.^10^^,^^11^ Athletes are commonly treated with ACL reconstruction (ACLR)^12^ and goal-directed rehabilitation programs for a successful return to sport. Despite these efforts, fewer than 30% to 50% return to the same sport level within 12 to 24 months after ACLR.^13–15^ Although previous research attributes an unsuccessful return to sport to various factors (eg, older age, poorer subjective rating of knee function, worse physical functioning), a prominent psychological factor is fear of movement, termed kinesiophobia, and in this context, particular fear of re-injury.^16^^,^^17^

An underlying cause for this fear may be that sports participation requires the athlete to perform various maneuvers that may involve ACL injury risk situations. Therefore, individuals with a high fear of re-injury might adopt specific movement strategies as an attempt to increase knee control. Such strategies are likely characterized by stiff movements with a restricted range of motion^18^^,^^19^ accompanied by muscle co-contraction around the knee joint.^20^^,^^21^ Higher fear of re-injury^22^ and higher kinesiophobia^23^ have been associated with a greater risk of secondary ACL injury, and biomechanical studies might shed further light on this association.

However, the existing research regarding associations between kinesiophobia and movement patterns among individuals with ACLR is currently limited to 2 studies, investigating gait^24^ and 2-legged drop-landings followed by a maximal vertical jump.^19^ There were no significant associations between kinesiophobia and gait parameters or knee kinematics or kinetics during gait.^24^ However, higher kinesiophobia correlated with lower peak flexion angles of the trunk, hip, and knee for the drop-landing task.^19^ These authors also found a significant association between kinesiophobia and greater preparatory gluteus maximus electromyography (EMG) activity before landing, but not for preparatory knee and ankle muscle activation patterns before landing or any lower limb muscle activation patterns during landing.^19^ Further research is warranted on the association between fear of re-injury and movement patterns during sport-mimicking maneuvers that emphasize the potentially hazardous side-to-side movements.^10^^,^^11^ Such associations would strengthen the view of fear of reinjury as an independent outcome of importance to consider throughout rehabilitation post ACLR to facilitate the return-to-sport transition and minimize secondary ACL injury risk.

We aimed to determine whether fear of re-injury was manifested in joint kinematics and muscle activation patterns during single-leg standardized rebound side-hop (SRSH) landings in individuals with ACLR with high fear compared with low fear and compared with asymptomatic non-injured controls. We also aimed to evaluate the subjective impact of fear of re-injury by comparing patient-reported outcome measures (PROMs) of knee function, knee health, and physical activity between the groups. We hypothesized that individuals with ACLR with higher fear would display a more defensive movement strategy with less joint motion and greater muscle activity in preparation for and during landing and accompanied by poorer PROMs than lower fear individuals with ACLR and asymptomatic controls. 

## Methods

### Design

This study was a cross-sectional observational study approved by the regional ethical review board in Umeå, Sweden (Dnr. 2015/67-31). Before participating, all participants provided written informed consent and agreed with the declaration of Helsinki. 

### Participants

Thirty-eight physically active individuals with ACLR within the last 2 years, stratified into either HIGH-FEAR or LOW-FEAR subgroups (see below), and 39 asymptomatic controls (CTRL) were included (Tab. 1). Participants were tested at the U-motion laboratory, Umeå University, Sweden, following a clinical knee examination by an experienced physical therapist. Individuals with ACLR were prospectively recruited over a period of roughly 2 years, mainly from the orthopedic clinic of the regional hospital but also in a few cases from a local sports medicine clinic and advertisements around the university and hospital campus. The following were inclusion criteria: 17 to 34 years of age, unilateral ACL injury, returned to physical activity and feeling confident in performing hop tests (ie, all individuals performed single-leg hops during their post-surgical rehabilitation), ipsilateral hamstring graft (standard practice nationally; >90%–95%, and to avoid mixed groups),^25^ no complete tear of any other knee ligament, no major menisci or articular damage, and no severe ankle sprain the last 6 months or other musculoskeletal/neurological pathology that would affect test performance. Similar relevant criteria were applicable for the controls recruited from advertisements at the university and hospital campus and by word of mouth. The control group was included to provide a reference of what would be considered a normal movement pattern in the current task among asymptomatic active individuals. All individuals with ACLR suffered their ACL injury during sports participation (30 non-contact, 4 indirect-contact, 4 contact) except for 1 individual (low-speed moped accident, knee forced in valgus and rotation). 

**Table 1.** Group Characteristics^a^

	HIGH-FEAR	LOW-FEAR	CTRL	*P*
Characteristic	N = 21	N = 17	N = 39	Main Effect
Male:female, n	10:11	8:9	7:32	–
Age, y, mean (SD)	24.0 (3.9)	25.5 (5.8)*^b^*	22.4 (3.0)	**.024**
Months after injury, median (IQR)*^c^*	16.0 (14.2–22.3)	18.0 (12.4–24.0)	NA	.894
Months after surgery, median (IQR)	11.2 (9.5–13.3)	10.1 (7.6–17.2)	NA	.509
Body height, m, mean (SD)	1.73 (0.09)	1.76 (0.08)	1.71 (0.07)	.369
Body mass, kg, mean (SD)	72.3 (10.3)	74.6 (12.2)	65.6 (7.8)	.070
Body mass index, kg/m^2^, mean (SD)	23.9 (2.2)	24.0 (3.2)	22.5 (2.0)	.150

*^a^* Bold *P* value indicates a significant effect (followed by Bonferroni post hoc) at the .05 level. CTRL = asymptomatic controls; HIGH-FEAR = individuals with ACLR with high fear of re-injury; IQR = interquartile range; LOW-FEAR = individuals with ACLR low high fear of re-injury.

*^b^* Significantly different from CTRL.

*^c^* Data are missing for 2 individuals in HIGH-FEAR and 3 individuals in LOW-FEAR because of uncertain estimates due to multiple knee pain scenarios before meeting a physician.

### Procedure

#### Patient-Reported Outcomes Measures

All participants filled out the PROMs before the hop testing and in the following order: 2000 International Knee Documentation Committee subjective knee form, Knee injury and Osteoarthritis Outcome Score, Lysholm Scale, International Physical Activity Questionnaire, Tampa Scale for Kinesiophobia-17 (TSK-17, only individuals with ACLR), and Tegner Activity Scale (pre-injury, current). 

#### Classification of Fear of Re-injury

The TSK-17 is a 17-item scale commonly used to estimate movement-related fear.^19^^,^^24^^,^^26^ This PROM was originally developed to “discriminate between non-excessive fear and phobia among patients with chronic musculoskeletal pain.”^27^ To specifically address the fear of re-injury, we used 1 statement from the TSK-17, Q9: “I am afraid that I might injure myself accidently,” which has 4 response options: “strongly disagree,” “disagree,” “agree,” and “strongly agree.” We used the 2 low-fear answers and the 2 high-fear answers to stratify the individuals with ACLR into subgroups of either LOW-FEAR (n = 17) or HIGH-FEAR (n = 21). We chose to use only the Q9 from TSK-17 to classify fear of re-injury to avoid other psychological aspects of fear and phobia (particularly pain) addressed by TSK-17. Further, because some of the items on TSK-17 refer to a person’s perception of what should be generalized as a rule for people suffering from their condition (eg, “It’s really not safe for a person with a condition like mine to be physically active”), these items may not be optimal in reflecting individual fear of re-injury. 

#### Hop Testing

The SRSH is a recently published single-leg hop test developed for biomechanical evaluation.^28^ In short, participants hopped barefoot on 1 leg from 1 force plate to another force plate in the ipsilateral direction, with respect to the performing leg, over a distance of 25% of body height, followed by an immediate rebound back to the starting position (see Suppl. Video). Participants held a 25-cm-long rope with both hands behind their backs to avoid obstructing markers and emphasize lower limb control. In addition, restricted arms result in greater knee joint valgus loading (thus suggesting a possibility of a higher risk of ACL injury/re-injury),^29^ greater knee joint work,^30^ and an increased difficulty to maintain balance at landing,^31^ all of which contribute to the task being more relevant in the context of fear of re-injury. The participants were allowed 1 or 2 familiarization trials before 5 successful trials were recorded. A successful trial required a 3-second single-leg stance after landing without letting go of the rope, putting down the contralateral foot, or shuffling the ipsilateral foot to maintain balance. 

### Biomechanical Assessment

#### Kinematics

Kinematics were registered using motion capture (8 cameras, 240 Hz, Oqus 300, Qualisys AB, Gothenburg, Sweden) and time-synchronized force plates (1200 Hz, Kistler Instrument AG, model 9260AA, Winterthur, Switzerland). A 6 degree-of-freedom model was constructed from 56 passive spherical markers attached with double-coated adhesive tape on the skin at anatomical landmarks, as previously described.^28^ Hip joint centers were defined using a functional joint method,^32^ with rigid clusters with 4 markers on the thighs (also used to reduce soft tissue artifacts^33^), and knee and ankle joint centers were derived from marker placements on femur epicondyles and malleoli, respectively.

Qualisys Track Manager (v.2.2, Qualisys AB, Gothenburg, Sweden) and Visual3D (v.5.02.19, C-Motion Inc., Germantown, MD, USA) were used for data processing and calculation of kinematic outcome measures. Marker data were filtered with a critically damped digital filter with a cut-off frequency of 15 Hz before calculating the dependent variables. The Cardan rotation sequence XYZ was used (X, mediolateral axis; Y, anteroposterior axis; Z, longitudinal axis).^34^ Trunk angles were defined relative to the vertical axis of the laboratory coordinate system, and hip and knee joint angles were determined from the movement of the distal segment relative to the proximal. Both angle and force data were then filtered using a fourth-order bidirectional 0-lag low-pass Butterworth digital filter with a cut-off frequency of 15 Hz. Kinematic outcomes investigated were sagittal and frontal plane trunk, hip and knee angles, and transversal plane hip and knee angles at initial contact (reflecting kinematics in preparation for landing)^19^ and range of motion during landing. 

#### Electromyography

Surface EMG was sampled at 1680 Hz (TeleMyo direct transmission system, model 542 DTS EMG sensor, Noraxon USA Inc., Scottsdale, AZ, USA) using silver-silver chloride, pre-gelled bipolar surface electrodes (Ambu BlueSensor N, Ballerup, Denmark) from biceps femoris, semitendinosus, vastus medialis, and vastus lateralis muscles. After shaving, scrubbing, and cleaning the skin surface with isopropyl alcohol, electrodes were placed over the muscle belly at an inter-electrode distance of 20 mm following SENIAM guidelines.^35^ The same test leader applied markers and EMG electrodes and instructed all participants.

**Figure 1:**
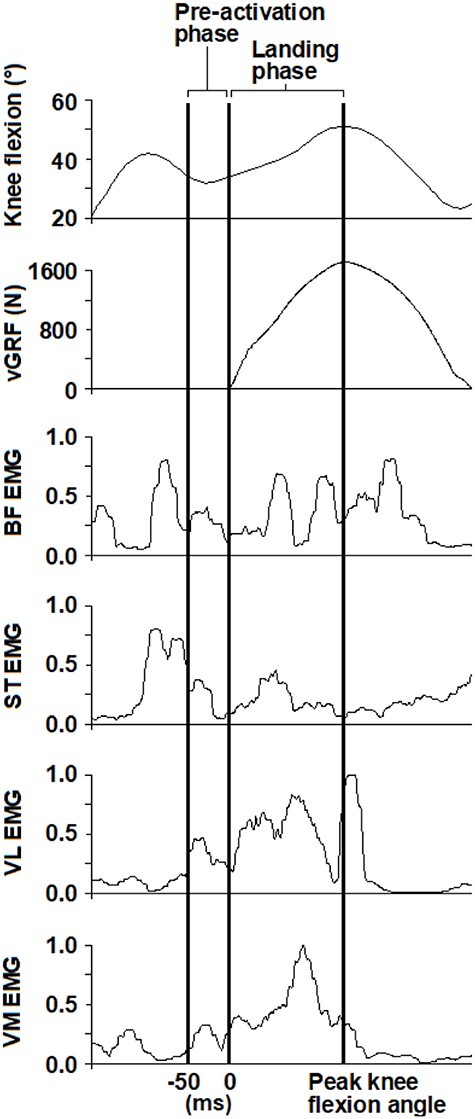
Data from 1 hop-landing trial for an individual with ACLR that displays the pre-activation phase, defined as a 50-ms phase prior to initial contact (at 0 ms, detected by the vertical ground reaction force [vGRF] > 20N), and the landing phase, defined as the period from initial contact until the peak knee flexion. EMG data that have been filtered, root mean squared processed, and normalized are shown for the 4 muscles of interest. BF = biceps femoris; ST = semitendinosus; VL = vastus lateralis; VM = vastus medialis.

EMG raw data were band-pass filtered (20 Hz–500 Hz) and applied a root mean square moving window of 20 milliseconds^35^ in Visual3D. Peak EMG values were extracted for each muscle and trial during the landing phase to calculate an average peak value that was used for normalization. This method is common^19^^,^^36^^,^^37^ and considers the force-velocity and length-tension relationships in muscles, often with higher EMG levels and less variability than in maximal isometric contractions.^38^ The average peak values were reliable with excellent intra-class correlations (model_(3,5)_, average measures form) of 0.90 to 0.99 for trial-to-trial peak values for all muscles in all groups. EMG outcomes investigated were mean amplitudes for each muscle and co-contraction indexes (CCIs) for anterior-posterior and medial-lateral muscles, both during a 50-millisecond pre-activation phase before initial contact (force plates registered loading >20N) and during the landing phase (from time of initial contact until time of peak knee flexion angle)^19^^,^^37^ (Fig. 1). CCI was calculated using the equation^37^:

CCI = (EMG_lower_/EMG_higher_)^*^(EMG_lower_+EMG_higher_),

where EMG_lower_ is the level of activity in the less active of flexor vs extensor (anterior-posterior CCI) or medial vs lateral muscles (medial-lateral CCI). 

### Missing Data

For 2 individuals in HIGH-FEAR, 1 individual in LOW-FEAR, and 3 individuals in CTRL, there were technical errors for EMG data for 1, 2, or all 4 muscles. These data were excluded from the EMG analyses. Data for kinematics and PROMs were analyzed for all individuals. 

### Statistical Analysis

The ACL-reconstructed legs in HIGH-FEAR and LOW-FEAR were compared with the non-dominant legs among CTRL (non-preferred leg when kicking a ball) for all between-group analyses in accordance with previous biomechanical studies from our research group.^39–42^ Mean values were calculated and used for each outcome variable from the 5 successful trials. Biomechanical outcomes and anthropometric group characteristics were normally distributed (Shapiro-Wilk test and histogram inspection). These outcomes were compared between groups with analyses of covariances with sex as a covariate to adjust for the different proportion of males and females in the groups. Significant results of analyses of covariances were followed by post hoc tests using Bonferroni correction. Effect sizes (ESs) eta squared were presented for main effects (classification: 0.01 = small, 0.1 = medium, 0.25 = large).^43^ PROMs were compared between groups using Kruskal-Wallis tests and, when significant, followed by Mann-Whitney pair-wise post hoc comparisons. The Statistical Package for the Social Sciences (v.25, IBM SPSS Statistics, Armonk, NY, USA) was used with *P* < .05 determining statistical significance. 

### Role of the Funding Source

The funders played no role in the design, conduct, or reporting of this study. 

## Results

### Fear in Relation to Kinematics During Landing

Differences between groups were revealed at initial contact for trunk flexion (*F*[2,73] = 3.7, *P* = .030, ES = 0.09 [small]), hip flexion (*F*[2,73] = 10.0, *P* < .001, ES = 0.22 [medium]), and knee flexion (*F*[2,73] = 14.6, *P* < .001, ES = 0.29 [large]), after controlling for sex. Specifically, HIGH-FEAR had more trunk flexion at initial contact than CTRL (*P* = .031), and both HIGH-FEAR and LOW-FEAR had more hip flexion (*P* < .001 and *P* = .011, respectively) and knee flexion (*P* < .001 for both) at initial contact than CTRL. No group differences were found for the other trunk, hip, or knee kinematic outcomes at initial contact or during landing (*P* > .05 for all) (Tab. 2). There were no significant differences in SRSH kinematics between the 2 ACLR subgroups classified according to fear of re-injury.

**Table 2.** Kinematic Data at Initial Contact and During Landing Presented in Mean (SD)*^a^*

	HIGH-FEAR	LOW-FEAR	CTRL	*P*
Kinematic Outcomes	N = 21	N = 17	N = 39	Main Effect
Angles at initial contact, deg				
Trunk flexion (+)	20.0 (4.5)*^b^*	18.7 (6.6)	15.5 (4.7)	**.030**
Trunk lateral bending (−)	−8.2 (3.4)	−6.5 (3.5)	−6.9 (2.5)	.178
Hip flexion (+)	41.7 (6.8)*^b^*	39.8 (6.2)*^b^*	33.9 (7.0)	**<.001**
Hip adduction (+)/abduction (−)	−12.2 (4.3)	−9.0 (5.2)	−10.7 (4.8)	.110
Hip internal (+)/external (−) rotation	5.2 (5.1)	4.5 (5.0)	4.1 (8.5)	.960
Knee flexion (+)	35.8 (7.6)*^b^*	37.9 (5.1)*^b^*	29.8 (6.0)	**<.001**
Knee adduction (+)/abduction (−)	−1.7 (3.6)	−3.6 (6.3)	−1.8 (5.8)	.372
Knee internal (+)/external (−) rotation	−11.1 (5.4)	−12.2 (3.8)	−10.1 (6.2)	.750
Range of motion during landing, deg				
Trunk sagittal plane	5.0 (3.2)	5.5 (3.0)	4.9 (3.2)	.854
Trunk frontal plane	3.0 (1.0)	3.7 (1.8)	2.9 (1.4)	.207
Hip sagittal plane	10.9 (5.6)	11.7 (5.0)	11.8 (6.4)	.521
Hip frontal plane	8.8 (4.3)	11.0 (4.2)	11.0 (4.7)	.095
Hip transversal plane	8.8 (2.7)	9.2 (2.4)	9.3 (3.1)	.933
Knee sagittal plane	25.7 (9.0)	27.3 (7.0)	28.8 (8.4)	.085
Knee frontal plane	10.3 (2.4)	10.9 (4.1)	10.7 (3.5)	.858
Knee transversal plane	9.4 (2.9)	10.9 (3.0)	10.2 (2.9)	.272

*^a^* Bold *P* values indicate a significant main effect after correcting for sex (followed by Bonferroni post hoc) at the .05 level. Positive values for flexion, adduction, internal rotation; negative values for lateral bending, abduction, external rotation. CTRL = asymptomatic controls; HIGH-FEAR = individuals with ACLR with high fear of re-injury; LOW-FEAR = individuals with ACLR with low fear of re-injury.

*^b^* Significantly different from CTRL.

### Fear in Relation to Muscular Activation Patterns During Landing

**Figure 2:**
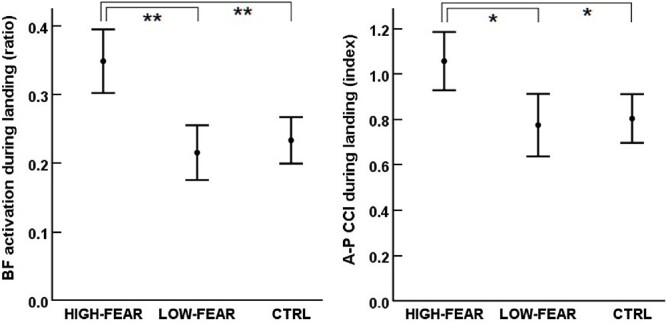
Muscle activation patterns that differed between groups with respect to fear of re-injury. Data show group mean values with 95% CIs. A-P CCI = anterior-posterior co-contraction index; BF = biceps femoris; CTRL = asymptomatic controls; HIGH-FEAR = individuals with ACLR with high fear of re-injury; LOW-FEAR = individuals with ACLR with low fear of re-injury. ^*^Statistical difference at .05 level; ^*^^*^statistical difference at .01 level.

Significant differences between groups were revealed for biceps femoris activation (*F*[2,71] = 14.6, *P* < .001, ES = 0.29 [large]) and anterior-posterior CCI (*F*[2,67] = 7.4, *P* = .001, ES = 0.18 [medium]) during landing. These outcomes indicated fear-related differences between the 2 ACLR subgroups. HIGH-FEAR had higher mean activation for biceps femoris and higher anterior-posterior CCI compared with both LOW-FEAR (*P* < .001 and *P* = .015, respectively) and with CTRL (*P* < .001 and *P* = .001, respectively) during landing (Fig. 2). No differences between groups were found for the other EMG outcomes in the pre-activation phase or during landing (*P* > .05 for all) (Tab. 3).

**Table 3.** EMG Amplitudes (in Ratio to Averaged Peak Values Across Trials) and Co-contraction Indexes During 50-ms Pre-Activation (Prior to Initial Contact) and Landing Presented in Mean (SD)*^a^*

	HIGH-FEAR	LOW-FEAR	CTRL	*P*
EMG Outcomes	N = 21	N = 17	N = 39	Main Effect
50 ms pre-activation				
BF mean amplitude	0.38 (0.12)	0.30 (0.11)	0.33 (0.14)	.152
ST mean amplitude	0.34 (0.14)	0.32 (0.13)	0.41 (0.17)	.469
VL mean amplitude	0.18 (0.09)	0.19 (0.09)	0.17 (0.08)	.265
VM mean amplitude	0.19 (0.09)	0.19 (0.07)	0.17 (0.05)	.212
Anterior-posterior CCI	0.57 (0.28)	0.56 (0.27)	0.48 (0.16)	.139
Medial-lateral CCI	0.90 (0.38)	0.80 (0.36)	0.84 (0.35)	.313
Landing				
BF mean amplitude	0.35 (0.10)*^b^*^,^*^c^*	0.22 (0.08)	0.23 (0.10)	**<.001**
ST mean amplitude	0.29 (0.10)	0.25 (0.08)	0.25 (0.08)	.172
VL mean amplitude	0.40 (0.09)	0.36 (0.05)	0.37 (0.09)	.194
VM mean amplitude	0.39 (0.08)	0.38 (0.06)	0.38 (0.08)	.537
Anterior-posterior CCI	1.06 (0.27)*^b^*^,^*^c^*	0.78 (0.26)	0.80 (0.32)	**.001**
Medial-lateral CCI	1.20 (0.22)	1.04 (0.17)	1.09 (0.29)	.051

*^a^* Bold *P* values indicate a significant main effect after correcting for sex (followed by Bonferroni post hoc) at the .05 level. BF = biceps femoris; CCI = co-contraction index; CTRL = asymptomatic controls; HIGH-FEAR = individuals with ACLR with high fear of re-injury; LOW-FEAR = individuals with ACLR with low fear of re-injury; ST = semitendinosus; VL = vastus lateralis; VM = vastus medialis.

*^b^* Significantly different from CTRL.

*^c^* Significantly different from LOW-FEAR.

### Fear in Relation to PROMs

The classification into HIGH-FEAR and LOW-FEAR using Q9 was corroborated by higher median scores for HIGH-FEAR for the TSK-17 total score (*P* = .003) and the TSK-17 subscale Activity Avoidance (Q1, Q2, Q7, Q9–12) both with (*P* = .001) and without (*P* = .038) Q9 that divided the groups (Tab. 4). Otherwise, there were no significant fear-related differences in PROMs between the 2 ACLR subgroups (*P* > .05 for all). Both HIGH-FEAR and LOW-FEAR had lower ratings for all PROMs than CTRL (*P* ≤ .001 for all), except for the Knee injury and Osteoarthritis Outcome Score Subscale Activities of Daily Living, where LOW-FEAR and CTRL had similar ratings (*P* = .240). Tegner Activity Scale ratings and International Physical Activity Questionnaire scores were similar between groups (*P* > .05 for all). Both HIGH-FEAR and LOW-FEAR groups reported lower current Tegner ratings than their pre-injury ratings (*P* ≤ .001 for all). 

**Table 4.** Patient-Reported Outcome Measures Presented in Median (IQR)*^a^*

	HIGH-FEAR	LOW-FEAR	CTRL	*P*:
Questionnaires	N = 21	N = 17	N = 39	Main Effect
TSK-17				
Total score, score 17–68	35.0 (30.0–36.5)*^b^*	28.0 (23.5–32.0)	NA	**.003**
Activity avoidance, score 7–28	13.0 (12.0–15.0)*^b^*	10.0 (8.0–12.0)	NA	**.001**
Activity avoidance (not Q9), score 6–24	10.0 (9.0–11.5)*^b^*	8.0 (7.0–10.0)	NA	**.038**
IKDC2000, % of maximum	77.0 (68.4–85.7)*^c^*	87.4 (78.2–92.6)*^c^*	100 (98.9–100)	**<.001**
KOOS, % of maximum				
Symptoms	78.6 (66.1–91.1)*^c^*	82.1 (76.8–94.7)*^c^*	100 (92.9–100)	**<.001**
Pain	86.1 (79.2–94.4)*^c^*	91.7 (87.5–97.2)*^c^*	100 (97.2–100)	**<.001**
Activities of daily living	98.5 (97.8–100)*^c^*	100 (98.5–100)	100 (100–100)	**.001**
Sports and recreational activities	70.0 (57.5–85.0)*^c^*	85.0 (77.5–97.5)*^c^*	100 (100–100)	**<.001**
Quality of life	56.3 (43.8–65.7)*^c^*	68.8 (59.4–75.0)*^c^*	93.8 (87.5–100)	**<.001**
Lysholm, score 0–100	84 (73–89)*^c^*	89 (85–95)*^c^*	100 (96–100)	**<.001**
Tegner activity scale, rating 1–10				
Pre-injury	8 (7–9)	8 (7–9)	NA	.927
Current	5 (4–7)	6 (4–7)	6 (4–8)	.849
IPAQ, total score	2807 (1934–4253)	3128 (2280–4813)	3488 (2488–4548)	.387

*^a^* Bold *P* values indicate a significant effect at the .05 level. CTRL = asymptomatic controls; HIGH-FEAR = individuals with ACLR with high fear of re-injury; IKDC2000 = 2000 International Knee Documentation Committee Subjective Knee Form; IPAQ = International Physical Activity Questionnaire; IQR = interquartile range; KOOS = Knee injury and Osteoarthritis Outcome Score; LOW-FEAR = individuals with ACLR low high fear of re-injury; NA = not available; TSK = Tampa Scale for Kinesiophobia.

*^b^* Significantly different from LOW-FEAR.

*^c^* Significantly different from CTRL.

## Discussion

Our findings revealed an association between muscle activation patterns, although not for kinematics, during sport mimicking SRSH landings to the reported level of fear of re-injury in individuals following ACLR. Individuals with high self-reported fear of re-injury displayed higher biceps femoris muscle activation and higher anterior-posterior CCI compared with both individuals with low fear of re-injury and asymptomatic controls. The classification of fear was corroborated by distinct scores between the ACLR subgroups for the TSK-17 total score and the subscale activity avoidance, although with no differences for PROMs addressing knee function, knee health, and physical activity. 

### Fear and Biomechanical Landing Patterns

Contrary to our hypothesis and the significant fear-related group differences for muscle activation patterns during the landing phase, we found no fear-related differences for any kinematic outcome or muscle activations during the 50-millisecond pre-activation phase. However, the higher reactive biceps femoris muscle activation and anterior-posterior CCI during landing in HIGH-FEAR compared with LOW-FEAR and controls suggest a knee protective strategy to stabilize the joint.^44^ These higher muscle activation patterns may result in a stiffer joint^20^ that in turn might serve to restrict anterior tibial translation to a greater degree, thus resulting in less strain on the graft.^36^^,^^45^^,^^46^ Notably, higher muscle co-contraction around the knee joint can result in increased tibiofemoral compression forces,^47^ which may have a detrimental effect as they potentially hasten the degeneration of the joint.^48^^,^^49^ Although non-significant, the trend of less knee range of motion in the sagittal plane during landing for those with higher fear of re-injury (HIGH-FEAR 25.7 degrees, LOW-FEAR 27.3 degrees, CTRL 28.8 degrees, *P* = .085) may additionally have increased the tibiofemoral compression forces, considering the function of the knee joint to act as a damper during impact. Consequently, our results may suggest that individuals with high fear of re-injury might expose themselves to a greater risk of accelerated knee joint osteoarthritis in the long term.

The only comparable study with ours that we know of that analyzed a sport-mimicking maneuver in relation to fear of re-injury (or kinesiophobia) analyzed the 2-legged drop-vertical jump test.^19^ These authors demonstrated that higher levels of kinesiophobia were associated with increased anticipatory EMG activity for gluteus maximus but not for knee and ankle muscle activation patterns either in preparation for landing or during landing. They also found significant associations with higher kinesiophobia and decreased peak trunk, hip, and knee flexion angles during landing.^19^ The discrepancy between these results and ours may be explained by task specificity. The instructions for the drop-vertical jump test were to jump as high as possible after impact, possibly resulting in a stronger emphasis on sagittal plane kinematics for individuals with high kinesiophobia, compared with the SRSH that had a lateral-to-medial hop. For the SRSH, rebounding back to the starting position does not imply a specific performance goal other than completing the task. Our results of higher reactive biceps femoris muscle activation and anterior-posterior CCI during the SRSH landing for HIGH-FEAR may relate to resisting frontal momentum, which is presumably not a priority during a 2-legged drop-vertical jump task. Furthermore, 2-legged landing tasks allow compensation using the other limb, as previously observed among individuals with ACLR.^50^^,^^51^ Further research is needed to investigate how fear of re-injury after ACLR affects movement patterns and how results vary between tests (eg, single-legged vs 2-legged, single-jumps vs repeated jumps, different directions). Additional knowledge of this association may be essential to facilitate the return to sport transition, considering that higher fear of re-injury^22^ and kinesiophobia^23^ has been related to an increased risk of secondary ACL injury. 

### Fear and PROMs

The lack of significant fear-related group differences for any of the PROMs (except those of the TSK-17) suggests that fear of re-injury is an independent outcome that does not necessarily relate to self-reported knee function and health among individuals with ACLR within the first 2 years post-surgery. However, this interpretation may only be valid for certain knee-demanding sport levels because both HIGH-FEAR and LOW-FEAR had lower current Tegner ratings (median of 5 and 6, respectively) compared with their pre-injury Tegner ratings (both median 8). Studies with larger sample sizes than ours (n = 135–1362)^13^^,^^15^^,^^52^^,^^53^ show that a higher level of kinesiophobia is associated with a lower return to recreational activity or sport, often with fear of re-injury reported as the main reason.^15^^,^^53^ How fear of re-injury relates to PROMs of knee function and health for individuals with different levels of physical activity certainly warrants further investigation. 

### ACLR-Related Biomechanical Landing Patterns Irrespective of Fear

Individuals with ACLR displayed different kinematics in preparation for SRSH landing compared with asymptomatic controls. The increased flexion angles observed at initial contact for both ACLR subgroups (at the trunk, hip, and knee joints for HIGH-FEAR, and hip and knee joints for LOW-FEAR) were possibly a preparatory strategy aimed to maintain knee control during landing. A flexion strategy results in a lower center of mass, which in turn assists in completing the task. Another reason for greater knee flexion angles at initial contact may be to protect the graft because it results in smaller patellar tendon insertion angles, greater hamstrings insertion angles, lower anterior tibial shear force, and lower tibiofemoral peak compression forces, all of which are considered to decrease ACL strain.^54–57^ Greater trunk and hip flexion angles also position the ground reaction force vector more anteriorly to the knee, thus reducing the knee flexion moment.^58^^,^^59^ Our results corroborate previous findings of a flexion strategy among individuals with ACLR compared with asymptomatic controls during hop testing.^21^^,^^58^^,^^60^ However, systematic reviews and meta-analyses also show no differences^61^ or less^62^ lower limb flexion angles during hop landings for individuals with ACLR compared with controls, so the results are inconclusive. Although flexion angles at initial contact did not differ between HIGH-FEAR and LOW-FEAR, these outcomes may be more suitably evaluated in a landing task with emphasized sagittal plane movement control, such as the drop-vertical jump.^19^

### Clinical Implications

Our findings link subjectively reported fear of re-injury to muscle activation patterns as suggestive of a knee-protective strategy. Fear of re-injury, evaluated in this case by a single question, constitutes a simple clinical screening tool that should be considered in relation to functional assessment (strength, hop performances) and biomechanical evaluation throughout rehabilitation for individuals with ACLR to facilitate the return to sport transition and to decrease the risk of a secondary injury. Our results also strengthen a recent consensus statement on sport-related injuries that recommends a holistic approach to optimize recovery^63^ by adapting biopsychosocial models to better manage fear-avoidance beliefs for physically active individuals.^64^ Also, future investigations of landing biomechanics among individuals with ACLR need to consider the fear of re-injury to avoid obscuring potentially essential findings. 

### Limitations

There is no gold standard measure of fear of re-injury following ACL rupture. The single question from TSK-17 that we used is not ACL specific, but the question (and all other PROMs) was answered considering the ACL injury. Our choice of only using the Q9 from TSK-17 to classify fear of re-injury should be considered when comparing our results with similar studies that used total scores for different TSK versions. Although the TSK-17 has been adapted to fit knee-injured patients better,^26^ the same issue with multiple psychological aspects remains. The sensitivity of using Q9 to classify individuals in groups of high- and low fear of re-injury was supported by the higher median scores for HIGH-FEAR for TSK-17 total score and the subscale Activity Avoidance (Tab. 4). However, further validation of merely using Q9 from the TSK-17 (or a similar question) is needed. Another limitation is that most controls were females, whereas we had an equal ratio of females and males in the ACLR subgroups. Although we adjusted for sex in the analyses, the higher number of female controls may still have resulted in a sex-specific bias for the biomechanical outcomes.^65^^,^^66^ Lastly, the variation in time between surgery and testing (min-max of 7–24 months) needs consideration because it does not allow a straightforward generalization to a specific time post-surgery. However, we found no correlations between time post-surgery to any biomechanical outcome, which could have affected the results. There were also no differences in time duration post surgery between HIGH-FEAR and LOW-FEAR. Therefore, we are confident that our fear-related findings can be generalized to individuals with ACLR who have returned to physical activity and sports. 

### Summary

Our findings show that individuals with ACLR with higher self-reported fear of re-injury exhibit atypical muscle activation patterns when performing single-leg SRSH. Fear of re-injury was associated with higher biceps femoris muscle activation and higher anterior-posterior co-contraction during landing yet poorly associated with anticipatory muscle activation patterns, kinematics, and self-reported knee function, knee health, and physical activity. Further, individuals with ACLR adapted, irrespective of fear of re-injury, a whole-body flexion strategy to prepare for landing compared with asymptomatic controls. Our study highlights that those individuals with ACLR should be evaluated for fear of re-injury throughout rehabilitation as a complement to functional assessment and biomechanical evaluation.

## Supplementary Material

PTJ-2020-0785_R1_SupplementaryVideo_pzab218

Supplementary_video_SRSH_pzab218
