# Supplementary material for: Fear of Reinjury Following Anterior Cruciate Ligament Reconstruction Is Manifested in Muscle Activation Patterns of Single-Leg Side-Hop Landings
Source: Phys Ther. 2021 Sep 21;102(2):pzab218. doi: 10.1093/ptj/pzab218 (PMC8860189; doi:10.1093/ptj/pzab218)
Supplement: PTJ-2020-0785_R1_SupplementaryVideo_pzab218 [file ptj-2020-0785_r1_supplementaryvideo_pzab218.pdf]

Supplementary Video

Title: A video showing one collected trial of the standardized rebound side-hop test.

Legend: Participants hopped barefoot in the ipsilateral direction with respect to the performing leg and immediately back again over a distance of 25% of their body height. They received instructions to stick the landing while aiming to regain control as fast as possible. The first landing from which the participants rebounded was analyzed. Participants held a 25 cm long rope with both hands behind their back to avoid obstructing markers on the body and legs and to emphasize lower limb control.
